# The sexual and reproductive health needs of young people living with HIV in Gauteng, South Africa

**DOI:** 10.4102/sajhivmed.v23i1.1377

**Published:** 2022-09-06

**Authors:** Bandile E. Ndlazi, Thembekile Masango

**Affiliations:** 1 Department of Health Studies, Faculty of Human Sciences, University of South Africa, Pretoria, South Africa

**Keywords:** sexual and reproductive health, HIV, young people, contraception, HIV disclosure

## Abstract

**Background:**

HIV has been the focus of health systems strengthening in South Africa for the past two decades. Despite progress, sexual and reproductive health (SRH) challenges such as contraception, condom usage and HIV disclosure of young people living with HIV (YPLHIV) remain inadequately addressed. Therefore, the purpose of the study was to describe the SRH needs of YPLHIV and make recommendations to address identified gaps.

**Objectives:**

To explore and describe the SRH needs and potential systemic gaps of YPLHIV with an aim to make recommendations for improvement and contribute to the development of an integrated approach to SRH care in HIV programming.

**Method:**

A quantitative cross-sectional research design with purposive sampling was utilised. YPLHIV were recruited from five healthcare facilities in Gauteng, South Africa, for face-to-face interviews.

**Results:**

One hundred and six YPLHIV with a median age of 18 years were enrolled. A large proportion (57/106; 53.8%) of respondents reported being either single or double orphaned. Sex-related discussions with parents were reported by only 36/106 (34.0%). History of teenage pregnancy was reported in 39/70 (56.0%) of female respondents. A high prevalence of multiple sexual partnerships 41/97 (42.2%) was noted. Consensual partner HIV disclosure was low at 47/97 (48.4%) and the male gender was associated with low 10/35 (28.6%) disclosure of serostatus to sexual partners.

**Conclusion:**

Multiple SRH needs were identified. Interventions are needed to improve parental guidance on SRH issues, increase contraception knowledge and access, and provide better male-centred care.

## Introduction

In 2021 it was estimated South Africa had 8.2 million people living with HIV.^1,2^ The HIV programme has made significant progress in South Africa, providing strong support for prevention, treatment, and care of those affected and infected. Almost 5.2 million people have been enrolled in the antiretroviral therapy (ART) programme and were receiving treatment in 2019.^3^ Availability of paediatric and adult ART has enabled the reduction of vertical transmission to less than 1% and the survival into adolescence and adulthood of many. A mathematical model study suggests approximately 400 000 children and adolescents are living with HIV in South Africa; this is attributed to high maternal HIV prevalence, but attenuated by the impact of ART through the prevention of mother-to-child HIV transmission programmes.^4^ The growth in numbers of children and adolescents living with HIV continues to create a gap in the parental understanding of their sexual and reproductive health (SRH) dynamics, which eventually has a negative effect on health-seeking behaviour and accessing of health services. As most young people living with HIV (YPLHIV) enter childbearing age, a clear understanding of their SRH needs, including dating, sex, contraception, condom usage, consensual partner serostatus disclosure and sexual practices is required, as is further exploration to devise effective interventions and develop focused guidelines.^5^

Social barriers, such as the perception and assumption that YPLHIV do not engage in sexual relationships due to their HIV status, serve to exacerbate limited access to relevant SRH information as it limits opportunities for sexual education and open discussions on sexual issues.^6^ YPLHIV desire to be accepted and live as their HIV negative peers, which may include engaging in risky sexual behaviours.^6^ Limited early access to sexual information and treatment further exposes YPLHIV to additional risks of HIV re-infection, opportunistic infections, and sexually transmitted infections (STIs).^7^ Deepened understanding of the SRH needs of YPLHIV will further inform the design of country-specific, culturally sensitive and multisectoral policies and programmes.^7^ Growing awareness of these challenges is gradually building more demand for healthcare services, especially those focusing on reproductive healthcare services for young people.^8,9^ As part of a holistic HIV treatment and care package, YPLHIV should receive culturally sensitive, accurate, and language- and age-appropriate health information on HIV management, STIs, contraception and safe sex. However, this seldom happens.^10,11,12^ Structural and social barriers are also still noted for adolescents to access SRH information, contraception, and other related services at an early age. Structural barriers refer to various cultural-driven norms that promote dominance of the older generation in making decisions directly affecting adolescents and young people, which further reduces young people’s opportunities for access to SRH services and related decisions.^13^

Our study aimed to describe the SRH needs of YPLHIV in order to contribute to the development of an integrated approach to this group’s SRH care in HIV programmes and guidelines.

## Research method and design

### Study design

A quantitative cross-sectional research design with the application of non-probability purposive sampling was utilised to study YPLHIV from five healthcare facilities in Gauteng, South Africa. Face-to-face interviews were conducted with respondents at the treating facility. The study applied a non-experimental design to explore questions pertaining to the SRH needs of YPLHIV and describe relationships among variables, rather than supporting inferences of causality.

### Setting

The study was conducted in three metropolitan districts in Gauteng, namely City of Ekurhuleni, City of Johannesburg, and City of Tswane. Non-probability convenience sampling was applied when selecting five healthcare facilities for the study. The selection was made conveniently with the direction of the district programme managers with the consideration of facility size, number of clients and the number of other studies in the facility, avoiding overburdening. The selected healthcare facilities were a mix of two community health centres and three primary health care facilities; all providing general preventative and curative services of minor ailments and chronic diseases including HIV prevention, treatment, care, and support. All professional nurses providing services to YPLHIV were trained on nurse initiated and management of antiretroviral treatment (NIMART) and, when required, patients are referred to doctors for further diagnosis and management.

### Study population and sampling strategy

Inclusion criteria were prospective respondents living with either vertically- or horizontally-acquired HIV and receiving HIV treatment, care and support from participating clinics, aged 18–24 years old, and willing to participate. Respondents’ HIV treatment adherence was not a prerequisite for this study. Mode of HIV acquisition was not used to determine eligibility. The Tier.Net electronic record system was used to determine the sampling frame from which the sample was drawn – no clinical data were extracted.

The sampling procedure was conducted to limit error and determine the sample size to assure reliable results. A sample size was computed from the total population of 374 young people aged 18–24 years receiving HIV treatment and care services in the five facilities drawn from Tier.Net. A representative sample size of 106 with a 5% margin of acceptance error was concluded for this study.


$$\begin{array}{l} \text{Desired sample size:}\\ \left(n)=\text{Population size }(\text{N})/(1+\text{total population}\times {\text{margin of error }(\text{e})}^{2}\right)\\ n=374/(1+374\times 0.05^{2})=106 \end{array}$$ [Eqn 1]

### Data collection

Data were collected between June 2018 and February 2019. The interviews were conducted by two trained research assistants using a standardised questionnaire adapted from John Cleland’s Illustrative Questionnaire for Interview-Surveys with Young People (see Appendix 2) to capture respondents’ responses to the assisted questionnaire with a maximum duration of 30 min. The list of prospective respondents drawn from Tier.Net was used to pull out the patients’ records to obtain their contact details. The research assistants approached those who were scheduled for their appointments and telephonically contacted those who were not scheduled for appointments. All respondents were asked to sign written informed consent. For standardisation, questionnaires were not distributed to respondents. The research assistants asked the questions and completed the questionnaires. Questions were translated into the respondent’s preferred language during the sessions. As respondents had already reached age 18, SRH historic questions were asked based on recall. All completed questionnaires were numbered according to the code assigned for the participating facility, with each respondent allocated an enrolment number for easy tracking of data errors and management of queries arising during data analysis. To maintain anonymity and confidentiality, only respondents’ codes (for identification by the researcher) and demographics were captured on the consent forms. Respondents with signs of distress and history of abuse were reported to facility managers for appropriate referrals.

### Data analysis

Raw data from completed questionnaires were coded, captured into an Excel^©^ data sheet, and crosschecked for accuracy and missing values. Data were then transferred to statistical software (Statistical Package for Social Sciences [SPSS]^©^ version 24) with the threshold for statistical significance set at *P* < 0.05 for all statistical analyses. Chi-square test was used to compare proportions. The statistical analysis plan included the generation of frequency distribution tables for all variables to enable exploration of data quality. Cross-tabulation was done to determine the relationship between the predictor variables and responses. Continuous variables were summarised using means with standard deviations or medians with interquartile ranges (IQR) as appropriate. Categorical variables were described as the total number and relative percentage of respondents per response category.

Participation was voluntary and open to all eligible prospective respondents without discrimination or coercion. The study objectives were the determining factor for inclusion and exclusion. Adolescents younger than 18 years were not included as the study was aimed at gathering data based on historical experiences. Prospective respondents’ culture, race and gender were not used to limit participation eligibility. The researcher treated the collected data and respondents’ personal information with strict confidentiality.

### Ethical considerations

The researcher obtained ethical clearance and permission to conduct the study from a Northern Gauteng university ethical review board (reference number HSHDC/630/2017). On receipt of the research proposal approval, approval was obtained from National Health Research Database (NHRD; reference number GP_2017 RP12_180). Application was submitted to the districts’ research committees for review and permission to access healthcare facilities, and approvals to proceed were granted on 15 February 2017.

## Results

A total of 106 respondents were interviewed in this study. The sample was predominantly female, 70 (66%). Respondents’ median age was 18 years (IQR: 17–19). All interviewed respondents were on ART – no information was collected on adherence and virologic outcomes. Detailed responses to the interview questionnaire are given in Appendix 1.

Data on HIV mode of transmission was self-reported. HIV acquisition was horizontal in 40 (37.7%), vertical in 56 (52.8%) and unknown in 10 (9.4%). Twenty-seven of the 56 respondents (48.2%) with vertically acquired HIV were informed of their positive HIV status when they were younger than 14 years. Education status was 44 (41.5%) respondents in Grade 10–12, 16 (15.1%) at tertiary level, and five (4.7%) in lower grades. Most of the study respondents self-reported being sexually active. Twenty-five (23.5%) respondents had left school before completing matric, eight due to pregnancy. Thirty-nine (55.7%) of the female respondents had a history of teenage pregnancy with eight reporting a miscarriage or stillbirth.

### General sex discussion at home

Fifty-seven (53.8%) respondents reported either both (*n* = 24/106 [22.6%]) or one (*n* = 33 [31.2%]) of their parents were deceased. Thirty of these 57 (52.6%) had been informed their parents’ cause of death was HIV. Sex-related discussions with parents were reported by only 36/106 (34%). Fifty-seven (53.8%) respondents had someone other than their parents with whom to discuss sex-related issues. Gender was associated with discussing sex-related matters with household members: more male respondents 30/36 (83.3%) reported having never discussed sex-related matters with others at home, compared to 40/70 (57.1%) of female respondents (*P* < 0.01).

### First consensual relationship partner’s profile and HIV disclosure to sexual partner

The first consensual relationship partner profile in the 86 respondents who reported these relationships was with someone 5–10 years older in 33 (38.4%) and was with someone more than 10 years older in four (4.6%). Five respondents (5.8%) knowingly had a relationship with a married person. The first consensual partners’ occupational status was 42 (48.8%) employed, 40 (46.5%) full-time students, and four (4.6%) unemployed.

Slightly above half, 50/97 (51.5%), had not disclosed their HIV-positive status to their sexual partners. Gender was associated with HIV status disclosure to sexual partners: 22/32 (68.7%) of the male respondents had never disclosed their HIV status to any of their sexual partners compared to 28/65 (46.1%) of the female respondents (*P* < 0.01). Reasons for HIV status disclosure are shown in Figure 1. Respondents who reported non-disclosure cited fear of rejection by their partners as the main reason.

**FIGURE 1:**
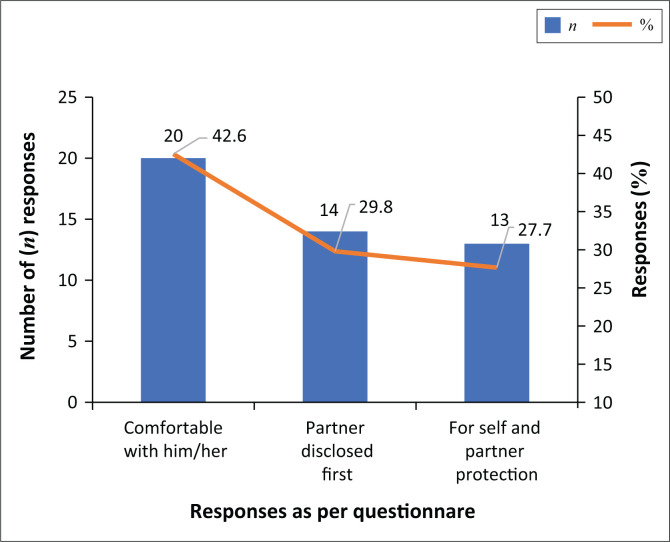
Reasons given for HIV serostatus disclosure in the 47 respondents who reported disclosure.

### Sexual practices

Sexual debut was reported by 97 respondents: 11 (11.3%) at 14 years of age, 27 (27.8%) at 15 years of age, and 57 at 16 years of age or older. The median age of sexual debut was 18 years (IQR: 16–19). Nine respondents (8.4%) reported to have never had sex. The first sexual encounter was consensual in 84/97 (86.5%), and non-consensual in 10/97 (10.3%). There was a high prevalence, 41/103 (41.8%), of multiple concurrent sexual partnerships while in a stable relationship.

Thirty-seven of 70 (52.8%) female respondents reported using pregnancy prevention methods. Nineteen of the 36 (52.8%) male respondents reported condoms as their contraceptive method of choice and six (16.7%) used withdrawal as a means of pregnancy prevention. Condom usage, practices, and beliefs are shown in Table 1. Self-reported condom usage at last sexual encounter was 84/95 (88.4%) while only 44/95 (46.3%) reported condom usage on sexual debut. Despite the high rate of condom usage among study respondents, findings revealed negative attitudes towards condom usage as 51/106 (48.1%) of respondents believed that condoms reduce sexual pleasure and 47/106 (44.3%) felt it was embarrassing for them to buy or collect condoms. Most female respondents 56/70 (80.0%) agreed women could proactively suggest the use of condoms, while more than half 20/36 (55.5%) of male respondents disagreed.

**TABLE 1:** Condom (both male and female condoms) usage, practices, and beliefs.

Response	Frequency	Total sample percentage	Valid % from responses
**Condom usage at last sexual encounter? (*n* = 95)**			
Yes	84	79.2	88.4
No	11	10.4	11.6
**Condom usage on first sexual encounter (sexual debut)? (*n* = 95)**			
Yes	44	41.5	46.3
No	51	48.1	53.7
**Condoms reduce sexual pleasure (*n* = 106)**			
Agree	51	48.1	48.1
Disagree	28	26.4	26.4
Don’t know	27	25.5	25.5
**Condom breakage during sex (*n* = 91)**			
Yes	47	44.3	51.6
No	44	41.5	48.4
**It is very embarrassing to buy or collect condoms? (106)**			
Agree	47	44.3	44.3
Disagree	48	45.3	45.3
Don’t know	11	10.4	10.4
**Suggesting the use of condoms indicates lack of trust (*n* = 106)**			
Agree	42	39.6	39.6
Disagree	62	58.5	58.5
Don’t know	2	1.9	1.9
**A girl/woman can also suggest the use of condoms? Women only (*n* = 70)**			
Agree	56	52.8	80.0
Disagree	22	20.8	31.4
Don’t know	2	1.8	2.8
**A girl/woman can also suggest the use of condoms? Men only (*n* = 36)**			
Agree	12	11.3	33.3
Disagree	20	18.8	55.5
Don’t know	4	3.7	11.1

Past STIs were reported by 44/106 (41.5%) with some admitting having had more than one recurrence. Most (42/44; 97.7%) respondents who reported STI incidence had sought medical attention, however only 23/44 (52.2%) notified their consensual partners.

### Healthcare-seeking behaviour and communication with healthcare workers

Sixty-four of 106 (60.4%) respondents indicated they were comfortable with asking clinic staff sex-related questions. Fifty-one of 64 (80.0%) respondents indicated they used HIV treatment follow-up consultation sessions as an opportunity to raise SRH-related questions, 53 of whom (82.8%) felt that their engagement with healthcare workers was fruitful as questions were responded to satisfactorily and without judgement. Gender was associated with whether the respondents felt comfortable with asking clinic staff questions: more male respondents (21/35; 60.0%) were not comfortable with asking clinic staff questions than female respondents (20/70; 28.6%) (*P* < 0.01).

## Discussion

The current study explored the SRH needs of YPLHIV. The study sample was predominantly female (66%) and included young people who have both vertically and horizontally acquired HIV infection. Teenage pregnancy was a common reason for females to drop out of school. These findings concur with Chakalisa et al., where 44% of female adolescents reported to have fallen pregnant during their adolescent years.^14^ High proportions of our respondents were single or double orphaned, which is important as orphaned children, more especially those growing up with vertically acquired HIV, become increasingly susceptible to a variety of hardships negatively impacting their quality of life.^15^ Integration of family planning and mental healthcare within HIV care could reduce unplanned pregnancies and other associated stressors among YPLIHV.^16^

The death of parents disrupts adolescents’ and young peoples’ quality of life and creates lifelong instabilities resulting in mental and behavioural challenges.^10^ Our findings that YPLHIV were more likely to have experienced the death of one or both parents is similar to those in a study from Cambodia where more than 50% of respondents reported at least one deceased parent.^9^ Ntuli et al. cited long-term effects of orphanhood, especially maternal death beyond age 18 years as these children suffer emotional distress from prolonged bereavement, which affects their ability to develop positive coping strategies.^17^ Their common coping strategy becomes silence and withdrawal which then further exacerbates their emotional distress.^17^

Absence of communication by parents and caregivers on sexual topics was found to be common in our respondents. Other studies have shown that parent-child sexuality discussions were described as selective, harsh and parent driven while children were just passive information receivers, and parents used threats to promote abstinence.^18,19^ Limited SRH support and guidance in the home has been linked to the perception and expectation that YPLHIV should not engage in sexual relationships.^6^ An Ethiopian study found only small proportions of young people reporting open communication with parents on topics pertaining to sex – only 28.0% were able to have related discussions with their mothers, and fewer, 18.5%, with their fathers.^20^

Sexual debut findings from the current study align with those of studies in Botswana and Zambia, where sexual debut was reported at 15 years of age.^14^ Generally, YPLHIV delay sexual debut due to their HIV status compared to the general population.^21^

The current study reported high condom usage at last sexual encounter, but very low condom usage at sexual debut. Contrary to these findings, low (38.0%) condom usage among sexually active YPLHIV was reported in a Ugandan study while 57.9% condom use at last sexual encounter was found in a study conducted among heterosexual sexually active young women in South Africa.^22,23^ The current study found inconsistent condom usage in most relationships, while convenience appeared to play an important role in condom usage with a relatively high proportion finding the purchase or collection of condoms embarrassing. Contrary to this finding, Wondemagegn et al. reported that only 5.0% referred to condom unavailability as a cause of inconsistent use.^24^ The current study’s findings, depicted high female autonomy with regard to condom suggestion and negotiation. The finding might not be a true reflection of the real practice: Ntshiqa et al. noted that young women reported low (47.0%) condom use with high risky sexual behaviour.^23^ In the current study, male respondents were more likely to engage in multiple sexual partners, aligning with those of other studies.^14,25^

Young men in the current study displayed reluctance regarding communication with healthcare workers regarding their SRH challenges; this was also noted in a study assessing the SRH service utilisation by male adolescents in a Gauteng district, where healthcare workers were perceived as judgemental.^26^ These findings indicate the need for male-focused SRH interventions.

Reported contraception use in this study was slightly lower than the national contraceptive use of 60.0% among sexually active women.^27^ A study conducted among women living with HIV in Soweto, South Africa, in 2010 found 84.0% were on contraception which is in contrast to our study; however, a study in Lao People’s Democratic Republic found only 34.0%.^28,29^ Different settings where data were collected could be the cause of data variations depending on other barriers. Withdrawal as a method to prevent pregnancy was reported by 16.7% of our male respondents which is twice as high as that reported by a study conducted in Lao People’s Democratic Republic.^29^

The current study found low consensual partner serostatus disclosure among YPLHIV. Our findings are similar to those of other studies.^21,22^ Male gender was associated with low serostatus disclosure to sexual partners compared to female in our study, which is consistent with other studies.^30,31^ These findings highlight the need for promotion of consensual partner disclosure of HIV and finding new approaches for partner notification, HIV index testing, and linkage to care and treatment. Early disclosure improves both the SRH and HIV treatment adherence and viral load suppression of YPLHIV.^32^

### Limitations of the study

Our study may be limited in generalisability to other districts and provinces because the sample was exclusively drawn from three metro districts, and because we only studied young people aged 18–24 years. We adopted a cross-sectional design and non-random sampling technique, which might pose a risk of bias. The mode of HIV acquisition relied on self-report.

## Conclusion

Our findings indicate multiple missed SRH needs of YPLHIV. Interventions are needed to improve parental guidance on SRH issues, contraception knowledge and access, especially directed at teenage pregnancies, reduction of HIV-related stigma, and provision of better male-centred care. A high proportion of YPLHIV are orphaned, which has profound impacts on health.

## Figures and Tables

**FIGURE 1-A2:**
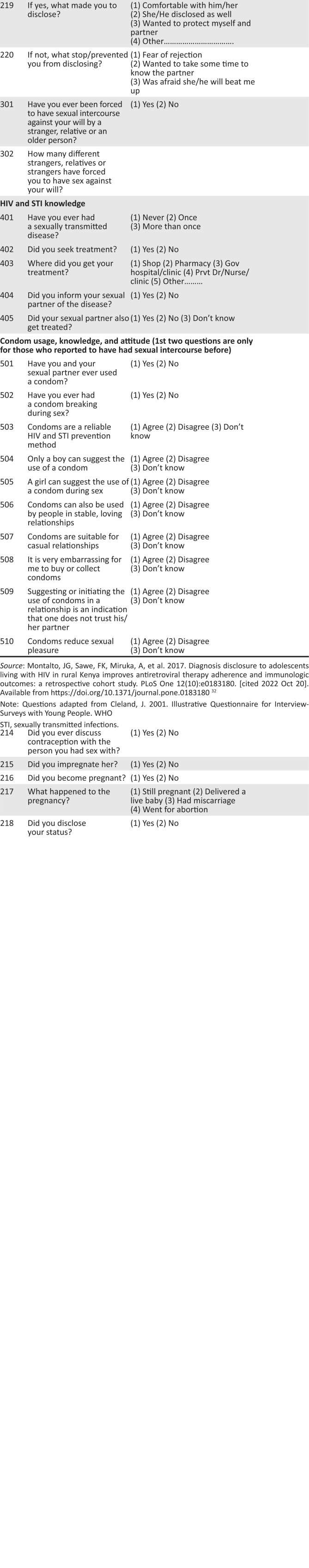
Interview questionnaire.

## Appendix 1

**TABLE 1-A1:** Responses to the interview questionnaire.

Responses to the interview questionnaire	Number (*n*)	Total sample (*n* = 106) (%)	Responses valid (%)
**Gender & age**			
Male (Median 18 years) IQR = 18–22	36	34.0	34.0
Female (Median 18 years) IQR = 19–23	70	66.0	66.0
Total (Median age 18: IQR = 18–19)	106	100.0	100.0
**Current level of schooling (*n* = 106/106; 100 %)**			
Grade 8–9	5	4.7	4.7
Grade 10–12	44	41.5	41.5
Tertiary level	16	15.1	15.1
Employed	10	9.4	9.4
Unemployed	31	29.2	29.2
**Last grade if left high school (*n* = 41/106; 38.7 %)**			
Grade 7	1	0.9	2.4
Grade 8	2	1.8	4.8
Grade 9	6	5.6	14.6
Grade 10	8	7.5	19.5
Grade 11	8	7.5	19.5
Grade 12	16	15.0	39.0
**Reasons for leaving or not completing school (*n* = 41/106; 38.7 %)**			
Completed Matric	16	15.0	39.0
Relocated after the death of parents and never pursued formal schooling	5	4.7	12.1
Pregnant	8	7.5	19.5
Was unable to attend classes due to recurring ill-health and hospitalisation	5	4.7	12.1
Poor financial support	7	6.6	17.0
**Was HIV contracted during pregnancy (from parents) (*n* = 106/106; 100 %)**			
Yes	56	52.8	52.8
No	40	37.7	37.7
Unknown	10	9.4	9.4
**Are your parents still alive (*n* = 106/106; 100 %)**			
Yes	49	46.2	46.2
No. Father died	22	20.8	20.8
No. Mother died	11	10.4	10.4
No. Both parents died	24	22.6	22.6
Total	106	100.0	100.0
**If any of your parents died, was HIV the cause of death (*n* = 57/106; 53.7 %)**			
Yes	30	28.3	52.6
No	19	17.9	33.3
Don’t know	8	7.5	14.0
**Who are you comfortable discussing sex-related matters with (*n* = 106/106; 100 %)**			
Mom	28	26.4	26.4
Dad	3	2.8	2.8
Both	2	1.8	1.8
Relative/Guardian	23	21.6	21.6
None	50	47.1	47.1
**Have you ever discussed any sex-related matters with any of your parents (*n* = 106/106; 100 %)**			
Yes	36	34.0	34.0
No	70	66.0	66.0
**Are you currently dating (*n* = 106/106; 100 %)**			
Yes	90	84.9	84.9
No	16	15.1	15.1
**How old is/was your first boy/girlfriend (*n* = 86/106; 81.1 %)**			
Same age as mine	24	22.6	27.9
Less than 5 years older	25	23.6	29.1
5–10 years older	33	31.1	38.4
More than 10 years older	4	3.8	4.6
**What was your first boy/girlfriend’s main activity when you started dating (*n* = 86/106; 81.1 %)**			
Full-time student	42	39.6	48.8
Employed	40	37.7	46.5
Unemployed	4	3.8	4.6
**Sexual debut (*n* = 97/106; 91.5 %)**			
14 years	11	10.4	11.3
15 years	27	25.5	27.8
16 years and older	57	54.8	58.7
**Contraception/Protection: Did you use a condom (*n* = 95/106; 89.6 %)**			
Yes	44	41.5	46.3
No	51	48.1	53.7
**Contraception: Are you on any pregnancy prevention methods, Female only. (*n* = 70/106; 66 %)**			
Yes	37	34.9	52.8
No	33	31.1	47.2
**Which pregnancy prevention method would you prefer your partner and you to use, Male only. (*n* = 36/106; 33.9 %)**			
Condom	19	17.9	52.8
Other hormonal contraception	11	10.3	30.5
Withdrawal	6	5.6	16.7
**Have you ever discussed contraception with any of the people you have/had sex with (*n* = 83/106; 78.3 %)**			
Yes	27	25.5	32.5
No	56	52.8	67.5
**Did you disclose your positive HIV status to any of your sexual partners (*n* = 97/106; 91.5 %)**			
Yes	47	44.3	48.4
No	50	47.1	51.5

IQR, interquartile range.
